# The peritoneal “soil” for a cancerous “seed”: a comprehensive review of the pathogenesis of intraperitoneal cancer metastases

**DOI:** 10.1007/s00018-017-2663-1

**Published:** 2017-09-27

**Authors:** Justyna Mikuła-Pietrasik, Paweł Uruski, Andrzej Tykarski, Krzysztof Książek

**Affiliations:** Department of Hypertensiology, Angiology and Internal Medicine, Poznań University of Medical Sciences, Długa 1/2 Str., 61-848 Poznan, Poland

**Keywords:** Cancer metastases, Peritoneal cavity, Reactive stroma, Seed and soil theory

## Abstract

Various types of tumors, particularly those originating from the ovary and gastrointestinal tract, display a strong predilection for the peritoneal cavity as the site of metastasis. The intraperitoneal spread of a malignancy is orchestrated by a reciprocal interplay between invading cancer cells and resident normal peritoneal cells. In this review, we address the current state-of-art regarding colonization of the peritoneal cavity by ovarian, colorectal, pancreatic, and gastric tumors. Particular attention is paid to the pro-tumoral role of various kinds of peritoneal cells, including mesothelial cells, fibroblasts, adipocytes, macrophages, the vascular endothelium, and hospicells. Anatomo-histological considerations on the pro-metastatic environment of the peritoneal cavity are presented in the broader context of organ-specific development of distal metastases in accordance with Paget’s “seed and soil” theory of tumorigenesis. The activity of normal peritoneal cells during pivotal elements of cancer progression, i.e., adhesion, migration, invasion, proliferation, EMT, and angiogenesis, is discussed from the perspective of well-defined general knowledge on a hospitable tumor microenvironment created by the cellular elements of reactive stroma, such as cancer-associated fibroblasts and macrophages. Finally, the paper addresses the unique features of the peritoneal cavity that predispose this body compartment to be a niche for cancer metastases, presents issues that are topics of an ongoing debate, and points to areas that still require further in-depth investigations.

## Introduction

Carcinogenesis is an extremely complex and mysterious disease, and its most critical and still insufficiently understood aspect is the separation of cancer cells from a primary lesion and their multistage journey towards various distant organs that eventually become colonized and give rise to the formation of secondary (metastatic) tumors. It has been estimated that as much as 90% of deaths in patients suffering from cancer is caused by a metastatic disease [1]. According to the current state-of-art, the pattern of metastasis distribution, which is considered a specific feature of a given cancer type, is determined by two complementary but plausibly not overlapping processes: mechanical (and rather passive) cancer cell dispatch by the lymphatic and/or venous systems followed by active colonization of the target tissue in accordance with Paget’s “seed and soil” theory [2].

The list of anatomical regions that serve as homing spots for secondary tumors is long; bones, for instance, are colonized mainly by breast and prostate cancer, and to a lower degree by lung, colon, thyroid, and bladder cancer. The brain, in turn, is the site of metastasis for melanoma, breast, lung, and colon cancer. Lung metastases are common in melanoma and breast cancer, whereas a spread within the liver occurs primarily in patients suffering from colorectal and pancreatic cancers [3]. Finally, the peritoneal cavity is a preferential site for metastasis of ovarian malignancy, albeit less often also other tumors, particularly those originating from the gastrointestinal system, give rise to intraperitoneal metastases [4].

Among all the above-mentioned organs serving clinically as metastatic niches, knowledge about the cellular and molecular determinants of peritoneal carcinomatosis seems to be the most enigmatic. At the same time, it still expands and provides certain conceptual challenges.

## Seed and soil theory of carcinogenesis

According to a classic and currently considered a very simplistic view, tumor development was the result of the accumulation of a significant number of oncogenic mutations. These abnormalities were placed within genes involved in cell cycle progression, apoptosis, and telomerase activity [5]. Unexpectedly, when this theory was already well rooted in the minds of scientists and clinicians alike, it turned out that immortal cells bearing a high number of oncogenic mutations are frequently unable to form tumors upon their transplantation into a laboratory animal’s body in vivo [6].

Stephen Paget, an English surgeon, was the first to propose that metastatic homing of malignant cells is not a stochastic event but, conversely, is governed by interaction between metastatically competent cancer cells (the “seed”) and the permissive microenvironment of specific organs (the “soil”). In consequence, successful cancer cell implantation in a distant location is possible only when cancer cells predetermined to spread throughout an organism will accept a special kind of molecular invitation sent by certain organs [2].

Paget’s theory was initially critically accepted, as other researchers had their own concepts in this regard; for instance, Ewing postulated that metastasis is determined by factors of a mechanical nature that are closely related to the unique vascular characteristics of a given region [7]. Others, e.g., Sugarbaker [8], presented a more balanced opinion and hypothesized that locoregional cancer spread results from both anatomical and mechanical determinants, whereas distant metastases are truly organ-specific.

Nonetheless, current knowledge on the mechanisms by which cancer cells colonize tissues has confirmed that although some anatomical predispositions, indeed, do matter, the organ-specific pattern of metastasis is primarily underlined by molecular compatibility between invading cancer cells and the tumor-accepting localization [9]. One of the best examples of this concerns breast cancer cells whose predilection to metastasize to the lymph nodes, bone marrow, lungs, and liver is determined by chemotactic interaction between malignant cells expressing chemokine receptors CCR7 and CXCR4 and tissues generating a high level of chemokine ligands for these receptors, i.e., CCL21 and CXCL12 [10].

Another example is the dissemination of melanoma cells when malignant cells administered intravenously metastasized to experimental pulmonary grafts and omitted control renal transplants [11]. Prostate cancer, in turn, preferentially colonizes the bones [12], which is attributed to the chemotactic activity of bone secretome products [13]. Last but not least, it is worthy to mention about the predilection of serous ovarian cancer to the peritoneal cavity which remained the prime site of metastasis even in patients treated with peritoneovenous shunts [14].

There is evidence that the capacity of certain distant locations to attract specifically cancer cells can be prepared remotely by factors released by primary tumors, e.g., vascular endothelial growth factor (VEGF), transforming growth factor β (TGF-β), and tumor necrosis factor α (TNFα) [15]. Various stimuli released by cancer cells mobilize bone-marrow-derived hematopoietic progenitors whose arrival to certain tissues determines very early changes in the local milieu, termed the “premetastatic niche” [16]. Organ-specific tumor metastases are also controlled at the genetic level by a wide array of transcripts that either provide some growth advantages in the primary and secondary locations or predispose to vigorous tumor expansion only in strictly specific sites [17].

The contemporary interpretation of the classic “seed and soil” theory assumes that the bidirectional crosstalk between cancer cells and the host tissue consists of several processes, e.g., invasion (inside and outside the circulation as well as into the tissue stroma), cancer cell adhesion to normal cells, migration towards a chemotactic gradient, and proliferation in response to autocrine and paracrine growth stimuli. Moreover, it also includes some additional and supportive but equally essential phenomena, e.g., the modulation of an immune response in the blood and target tissue, epithelial–mesenchymal transition (EMT), mesenchymal–epithelial transition (MET), and angiogenesis [18, 19]. Several of the above-mentioned processes underlying the formation of a metastatic niche are governed by extracellular matrix (ECM) constituents, periostin, and tenascin C, that activate Wnt and Notch pathways in cancer cells, providing both physical and signaling support for cells that initiate a metastasis [20, 21]. Now, this complex functional network, shaped and regulated to a significant degree by normal cells neighboring the malignancy, is called the “reactive stroma”. This term emphasizes that the cancer-accepting tissue is not a passive recipient of the cancer cells but is instead an active player governing the most critical elements of the disease.

## Reactive, cancer-associated stroma

The tumor stroma consists of distinct cell types whose heterotypic interactions with malignant cells and one another drive tumor progression. At the moment, the most appreciated peritumoral representatives of this structure are cancer-associated fibroblasts (CAFs) [22] and tumor-associated macrophages (TAMs) [23].

### Cancer-associated fibroblasts

The unique properties of CAFs were first reported in 1999 by Olumi and colleagues, who found that fibroblasts isolated from prostate cancer are able to, as opposed to cells from a noncancerous gland, initiate the malignant transformation of prostate epithelial cells and the growth of tumors in immunocompromised animals [24]. Further research using cells from invasive mammary cancer allowed to define CAFs as cells: (1) with explicit tumor-promoting activity, (2) containing a large fraction of α-smooth muscle actin (αSMA)-positive myofibroblasts co-existing with fibroblasts resembling those from normal tissues, (3) with proangiogenic capabilities, i.e., associated with augmented secretion of CXCL12/SDF-1, which were greater than those characterizing normal fibroblasts, and (4) with the preserved capacity to promote tumors and exert myofibroblastic features even in the absence of cancer cells [25]. Thanks to their ability to secrete cytokines (e.g., IL-6), chemokines (e.g., CXCL8/IL-8), growth factors (e.g., FGF, HGF, TGF-β, VEGF), and extracellular matrix proteins, and remodeling enzymes (e.g., collagen I, tenascin C, periostin, fibronectin, MMP-1), CAFs literally support all vital steps of tumor progression. Their contribution to carcinogenesis extends from the conversion of pre-malignant cells to full-blown malignancy to the final formation of distant metastases [26]. Interestingly, in some cases, the presence of CAFs may also have some positive aspects. This applies, e.g., to pancreatic adenocarcinoma where a depletion of CAFs initiated immunosuppression and reduced patient survival [27].

One of the best recognized mechanisms by which CAFs contribute to cancerogenesis is TGF-β-related signaling. The activity of TGF-β seems to be critical in the very initial phases of tumor formation due to its profound immunosuppressive activity [28]. It has been found that CAFs determine the propensity of adjacent epithelia (prostate and forestomach) to be oncogenic in the TGF-β-dependent mechanism [29]. Similar observations were made using colorectal cancer cells whose efficiency for organ colonization was positively regulated by stromal cell-derived TGF-β, and animals subjected to the pharmacological inhibition of TGFBR1 appeared to be resilient to metastasis formation [30]. The pro-metastatic effects of TGF-β were further mediated by anti-apoptotic GP130/STAT3 signaling and the GP130 ligand, interleukin-11 (IL-11), which is produced exclusively by CAFs in response to TGF-β. The remaining, already identified down-stream pro-metastatic effectors of this cytokine include connective tissue growth factor (CTGF) [31], tenascin C (TNC) [32], and angiopoietin-like 4 (ANGPTL4) [33]. These molecules contribute to metastasis formation using various routes. CTGF induces hypoxia-inducible factor 1α (HIF-1α)-dependent reprogramming of CAFs that leads to the activation of tumor-supporting autophagy, glycolysis, and senescence [34]. TNC promotes cancer cell survival, proliferation, migration, and EMT [35], whereas ANGPTL4 contributes mainly to increased angiogenesis [33].

It is worth noting that the activity of TGF-β in a tumor microenvironment is not solely pro-cancerous, per analogy to the activity of CAFs [26], e.g., mutations in the tumor suppressor gene *APC* combined with inactivation of TGFBR2 in epithelial intestinal cells enabled the malignant transformation and invasion of colorectal carcinoma in a mouse model [36]. We strongly believe that the activity of TGF-β in cancer is highly context-dependent; however, a detailed analysis of this dichotomy is far beyond the scope of this article (see [37–39] for excellent reviews of this topic).

Another interesting pathway by which CAFs appear to influence tumor development and progression is cellular senescence. In fact, senescent fibroblasts that are capable of initiating carcinogenesis [40] as well as of promoting cancer cell progression both in vitro and in vivo [41] have been considered as one of the probable sources of CAFs. The similarity between CAFs and senescent fibroblasts is in particular expressed in their ability to overproduce several pro-cancerous stimuli, which is called the senescence-associated secretory phenotype (SASP) [42]. Research on breast cancer cells revealed that senescent fibroblasts which are specific for sites of cancer metastasis promoted the growth of malignant cells thanks to their ability to hypersecrete interleukin 6 (IL-6), whereas cells that produced little to none of this cytokine failed to support tumor growth in the mouse xenograft model [43]. Interestingly, however, both senescent and nonsenescent CAFs appear to display diversified activity, as the former have been found to support aggressive cancer phenotypes more efficiently [44]. Simultaneously, there is evidence that sometimes, the activities of CAFs and senescent fibroblasts do not overlap. This is the case, for example, for gastric fibroblasts which upon treatment with IL-6 transdifferentiated into CAFs in a mechanism involving Twist1-dependent phosphorylation of STAT3. Although ectopic expression of Twist1 in normal cells inhibited their senescence, suppression of this transcription factor accelerated senescence in the CAFs [45].

### Tumor-associated macrophages

Taking into account that cancer in many aspects resembles a state of chronic inflammation [46], cells representing the immune system, and in particular macrophages, play an important role as active elements of the reactive stroma [47]. The recruitment of macrophages into tumors is mediated by cytokines, chemokines, and growth factors originating from cancer and nearby normal tissue stroma. The most important chemoattractants for these cells include CCL2, CCL3, CCL4, CCL5, and CCL22 [48]. Tumor-associated macrophages (TAMs), usually observed on the boundaries of a tumor, are classically linked with their ability to restrict the extent of damaged tissue through their ability to scavenge necrotic debris [49].

Another effect attributed to TAMs is immunosuppression directed mainly towards the T-cells. This capability is expressed exclusively by the M2 subtype of macrophages, mainly by the M2d cells [50]. These cells, in contrast to the M1 fraction bearing pro-inflammatory characteristics, have anti-inflammatory properties associated with the production of various molecules, including IL-10, TGF-β, and arginase 1 [51]. Moreover, the macrophages elicit T-cell dysfunction (depressed proliferation and cytotoxicity) through TNFα- and IL-10-dependent induction of programmed death-ligand 1 (PD-L1) [52]. Simultaneously, they have the ability to mobilize natural regulatory T-cells (nTreg), which proceed in a mechanism involving the chemotactic activity of CCL3, CCL20, and CCL22 [53].

One of the most intriguing features of TAMs is their functional switch related to the stage of tumor development. In the initial phases, macrophages infiltrating a tumor display the M1 phenotype and tend to eliminate the malignancy. As the pathology progresses, however, the macrophages adopt the M2 function (often described as IL-12^low^/IL-10^high^) and start to alter the microenvironment into a cancer-promoting phenotype [48].

TAMs also modulate further invasion of normal tissue by cancerous cells by secreting ECM-degrading enzymes, such as matrix metalloproteinases [54] and cysteine protease, cathepsin [55]. As per the metalloproteinases, TAMs usually operate through MMP-1, MMP-7, MMP-9, and MMP-12 [48]. When it comes to cathepsin, recent reports have suggested that massive tumor infiltration with macrophages followed by release of significant amounts of the enzyme occurs in mammary tumors upon the administration of paclitaxel. Macrophages expressing cathepsin protected the cancer cells against drug-induced death and this effect was effectively prevented by cathepsin inhibition. The same macrophages were also found to inhibit the incidence of cancer cell death elicited by etoposide and doxorubicin [55]. Mechanistically, the activity of cathepsin in TAMs is associated with the activation of autophagy, including the fusion of autophagosomes and lysosomes, leading to the development of the prototypic, polarized M2 phenotype in these cells [56].

## The peritoneal cavity: a brief look at structure and function

The human body consists of several cavities, of which the pleural, pericardial, and peritoneal cavities are the most important ones. Among these cavities, the peritoneum is the most extensive. The peritoneum has two layers—the parietal and the visceral layer. The parietal peritoneum covers the walls of the abdomen and pelvis, whereas the visceral peritoneum lines the coelomic organs. The space between these two layers, i.e., the peritoneal cavity, is in physiological conditions filled with a small amount (~ up to 100 ml) of fluid [57]. Under pathologic conditions (e.g., cancer), the fluid’s volume increases and its biochemical composition changes dramatically, which often correlates with poor prognosis [58].

From a histological point of view, the peritoneum consists of two general compartments, i.e., the mesothelium and the stroma. As opposed to the mesothelium, which is formed by a single layer of epithelial-like cells resting on a basement membrane, the stroma consists of both cellular (fibroblasts, macrophages, mast cells, and endothelial cells) and acellular elements (collagen, glycoproteins, and proteoglycans). An important structural component of the peritoneal cavity is adipocytes, which are particularly abundant within the greater omentum, where they form the visceral fat coat. The blood and lymphatic vessels as well as nerves are present in the subserous space [59].

Apart from being a framework where visceral organs are anchored and serving as a conduit for their vascularization and innervation, the peritoneal cavity has several additional functions whose realization is guaranteed by reciprocal interactions between the diversified populations of cells forming this cavity. The most classic function is lubrication of both the peritoneum surfaces which allows for frictionless movements of the viscera. This property is provided by the peritoneal mesothelial cells (PMCs), which have the constitutive ability to produce and release surfactant-like proteoglycans and phospholipids [57]. Another basic function of the peritoneum is the filtration, as the peritoneum is a semipermeable membrane for the bidirectional passage of water and dissolved particles between the blood and the peritoneal cavity [60].

Last but not least, the principal destiny of the peritoneal cavity is a contribution in certain forms of inflammatory reactions [61]. This activity is regulated by a network of paracrine and autocrine interactions between normal peritoneal cells and the products of their constitutive or inducible secretome. The first line of defense is the peritoneal macrophages (PMs), which have the ability to generate significant amounts of the tumor necrosis factor (TNF). Their activity is followed by reactions elicited by PMCs which secrete a plethora of soluble mediators to the environment, such as cytokines (IL-1, IL-6, IL-15), chemokines (CXCL8/IL-8, CCL2/MCP-1, RANTES, CXCL1/GRO-1, and CXCL12/SDF-1), growth factors (TGF-β1, PDGF, FGF, and VEGF), ECM elements (collagens I, III, IV, fibronectin, elastin, and vitronectin), and adhesion molecules (ICAM-1, VCAM-1, E-cadherin) [62]. An important activity of the mesothelium is also the generation of the chemotactic gradient for polymorphonuclear leukocytes, which is related to the secretion of interleukin 17 [63]. A supportive role with respect to PMCs is played by peritoneal fibroblasts (PFBs), which share with them the general profile of the secretome and also attract polymorphonuclear cells, but in an interleukin 1β (IL-1β)-dependent mechanism [64].

## Intraperitoneal carcinomatosis

The peritoneal cavity is attacked by different types of cancer cells, albeit the frequency and mechanisms by which malignant cells reach and colonize the peritoneum differ remarkably. Most frequently, the peritoneum attracts ovarian, colorectal, pancreatic, and gastric tumors. Less common are metastases of breast and lung cancer, as well as those from melanoma [65].

One of the most important features of the peritoneum that makes this organ an excellent site for the development of secondary tumors is its extensive area; the second feature is the presence and movement of the peritoneal fluid. When the ascites accumulate, starting in the pouch of Douglas and further in the other compartments of the peritoneal cavity, their flow gathers tumor cells and distributes them in, to some extent, a stochastic manner throughout the whole cavity. On the other hand, the fluid circulates in a well-defined manner (in the cephalad–caudal–cephalad direction and controlled by gravity and respiratory motion), which means that there are some locations with a particular propensity to deposit inflowing cells; these include the pouch of Douglas, the sigmoid colon and its mesentery, the terminal ileum, the right paracolic gutter, the posterior right subhepatic space, and the right subphrenic space [66].

Another common location of metastatic tumors is the greater omentum, which anatomically floats in the peritoneal cavity and is bathed by the peritoneal fluid. In the case of some malignancies, particularly ovarian cancer, the greater omentum is the most frequent place for metastasis [67]. Deposits of cancerous cells within the omental tissue have been found in as much as 46% of patients in stage III disease [68]. A special predilection of cancer cells to colonize the greater omentum is associated with the presence of adipose tissue-derived mesenchymal stem cells [69] as well as with the abundancy of milky spots [70]. Studies employing various types of cancers, e.g., melanoma, lung, breast, and ovarian carcinoma, showed that the peritoneal metastases of these tumors preferentially colonize omental milky spots consisting of organized aggregates of immune cells and a complex network of capillaries with a high vascular density [70].

The omental milky spots and omental adipocytes seem to exert complementary action towards the promotion of intraperitoneal tumors. This assumption stems from in vivo experiments which showed that various lines of ovarian cancer cells lodge and progress more preferentially within omental and splenoportal fat that is rich in milky spots than within peritoneal fat deposits. Moreover, a conditioned medium generated by adipose tissue with the milky spots promoted cancer cell migration more efficiently than the medium from adipose tissue lacking these structures [71].

### Ovarian cancer

Most often, the peritoneum is the site of homing for ovarian cancer cells. Peritoneal tumors have been found to be developed in as much as 70% of patients in stage III or IV of the disease [72]. Primarily, the predilection of the peritoneal cavity to attract ovarian cancer cells is dictated by the fact that the ovaries are suspended in the peritoneal cavity and that the ovarian epithelium constitutes a continuity with the PMCs [4]. The peritoneal spread of the primary ovarian tumor is thus a perfect example of direct intraperitoneal seeding. Ovarian cancer may also spread along the broad ligament to engage the serosal side of the uterus, or, alternatively, it may progress laterally to occupy the peritoneum of the pelvic sidewall [66].

The exfoliation of cancer cells from their primary location is accompanied by their morphological reorganization, in particular initiation of the EMT due to decreased expression of a membrane glycoprotein, E-cadherin [73]. Decreased expression of this protein results in the development of a spindle-shaped morphology of the cancer cells, which become more invasive. Moreover, down-regulated expression of E-cadherin correlates with an increased level of α_5_-integrins and results in increased adhesion of cancer cells to the three-dimensional omental culture consisting of PMCs and fibroblasts [74].

Once the cancer cells are successfully detached from the primary tumor and reach the peritoneal space, they are carried by the peritoneal fluid, which is usually present in excess in the form of malignant ascites [58], and then float passively to finally sediment on certain surfaces of the peritoneal cavity. To decrease the probability of elimination by intraperitoneal inflammatory cells, most cancerous cells form conglomerates, i.e., “spheroids”, in which they remain until final disaggregation takes place, announcing the initial phase of cancer cell adhesion to resident normal peritoneal cells [75]. Free-floating cells are still in the EMT state [76], which may be causatively linked with high expression of Sip1, which is a negative regulator of the E-cadherin level [77].

The peritoneal malignant ascites that constitute an environment for ovarian cancer cells act not only as their passive carrier but also actively contribute to progression of the disease. They modulate immune reactions within the peritoneal cavity, e.g., they inhibit T-cell receptor-induced NF-κB and the nuclear factor of activated T-cell (NFAT) signaling in tumor-associated T-cells [78]. In addition, the ascites are rich in soluble agents that support tumor growth and tissue neovascularization, including angiogenin, VEGF, IL-6, CCL2/MCP-1, CXCL1/GRO-1, and CXCL8/IL-8 [79]. A recent study revealed that this fluid’s biochemical composition, in particular the high concentration of several pro-inflammatory agents, may be responsible for the high aggressiveness of undifferentiated ovarian tumors [80].

As for the adhesion of ovarian cancer cells to surfaces of the peritoneum, in particular to PMCs and ECM proteins, it should be pointed out that this process is the first of several phenomena based on interactions between cancer cells and normal peritoneal cells whose ultimate goal is the formation of solid intraperitoneal metastases [4]. The disaggregation of spheroids allowing for the initiation of adhesion is related to the proteolytic activity of matrix metalloproteinase 2 (MMP-2) against fibronectin and vitronectin [81]. Further steps include migration of the cancer cells towards a chemotactic gradient generated by soluble stimuli released by the mesothelial cells, fibroblasts and adipocytes, invasion across the mesothelium, ECM and basement membrane to reach the tissue stroma, and, finally, proliferation, again fueled by soluble mitogens of different origin, which yields new generations of malignant cells that can form a tumor [4].

Apart from the hospitable “soil” provided by the peritoneum, the cancerous “seed” also actively helps to create a metastatic niche. A perfect example of this activity is TGF-β1/Smad 2/3-dependent signaling that is activated by transcription factor PITX2 which modulates ovarian cancer cell invasion [82]. Another example is the activity of cancer-derived exosomes that are rich in the CD44 molecule internalized further by the mesothelial cells that alter the phenotype of the latter towards the augmentation of certain cancer-promoting features (e.g., increased MMP-9 secretion) [83].

In addition, colonization of the peritoneal cavity is supported by cancerous neoangiogenesis [84], which is promoted in a clearly overlapping manner by malignant ascites [85] and the products of the normal [86] and malignant cells’ [87] secretome.

### Gastrointestinal cancers

Somewhat less often than in the case of ovarian cancer but still frequently enough to be a clinical problem, the peritoneum is a site for the dissemination of gastrointestinal (colorectal, pancreatic, and gastric) tumors. As per colorectal cancer, the peritoneum is the second, to the liver, distant location to be colonized by malignant cells [88]. Statistically, even 80% of patients who died from this pathology had intraperitoneal metastases [89]. Pancreatic cancer disseminates, in turn, within the liver and the peritoneum, where it develops tumors most frequently within the greater omentum [90]. It has been estimated that 70–80% of nonresectable patients with pancreatic tumors experienced peritoneal carcinomatosis [91]. Finally, when it comes to gastric cancer, up to 50% of patients with advanced disease develop peritoneal tumors, even despite radical surgery [65].

Peritoneal involvement is also a sign of disease recurrence. It has been found that in up to 35% of patients with colorectal cancer and in up to 50% of patients with gastric cancer, cancer recurrence was confined to the peritoneal cavity. In contrast, however, to ovarian tumors where cytoreductive surgery followed by elimination of focal microtumors using chemotherapy results in disease recurrence in the relatively long perspective, the recurrence of gastrointestinal tumors is fast even upon total eradication of their metastases from the peritoneum [65].

From the pathophysiological point of view, peritoneal dissemination of gastrointestinal cancers typically proceeds in two ways, i.e., as a result of direct cell detachment from a primary tumor (along with bowel wall penetration in the case of colorectal cancer) or iatrogenically due to incomplete resection of the primary lesion and cancerous cell efflux from dissected blood and lymph channels [89]. If the cancer cells are detached spontaneously, they are pushed by the high pressure of the interstitial fluid to seed within the peritoneal cavity. Some factors increase the interstitial pressure; these include contraction of the interstitial matrix, tissue fibrosis, osmotic pressure elicited by anaerobic glycolysis, and the escape of plasma proteins [92].

Once the cancer cells of gastrointestinal origin get to the peritoneum, their implantation in the metastatic niches requires, again, their strict cooperation with normal peritoneal cells. The essence of adhesion, migration, and invasion as well as of EMT and angiogenesis is analogical to that described for ovarian cancer cells [92]. In some cases, however, e.g., during adhesion, the mediators of both cancer cell and normal cell origin are different. It should be emphasized that the dialogue between cancerous and normal cells proceeds in both directions, which means that the cancer cells are also actively engaged in the colonization process. This activity has been shown when analyzing the movement of colorectal cancer cells towards tissue stroma whose process takes place through gaps between the PMCs which were likely formed in response to the pro-apoptotic signals of cancerous origin [93]. An important role is also played by malignant ascites; e.g., MMP-7, which, present in the fluid in patients with gastric cancer, appeared to be predictive of peritoneal cancer spread [94].

### Tumors metastasizing using the hematogenous and lymphatic route

Hematogenous spread into the peritoneal cavity is encountered in patients with malignant melanoma, lung, and breast cancer. In such cases, the embolic metastatic focus begins as a small nodule with eventual progression. The lymphatic dissemination involves, in turn, channels that are common along the ligaments and mesenteries within the peritoneal cavity. This leads to the formation of round and/or oval tumors and occurs particularly in patients with nonHodgkin’s lymphoma. Current appreciation of this kind of peritoneal involvement is to some extent underestimated, as this form of transmission plays a clinically negligible role [95].

## Cellular elements of cancer development within the peritoneal cavity

**Fig. 1:**
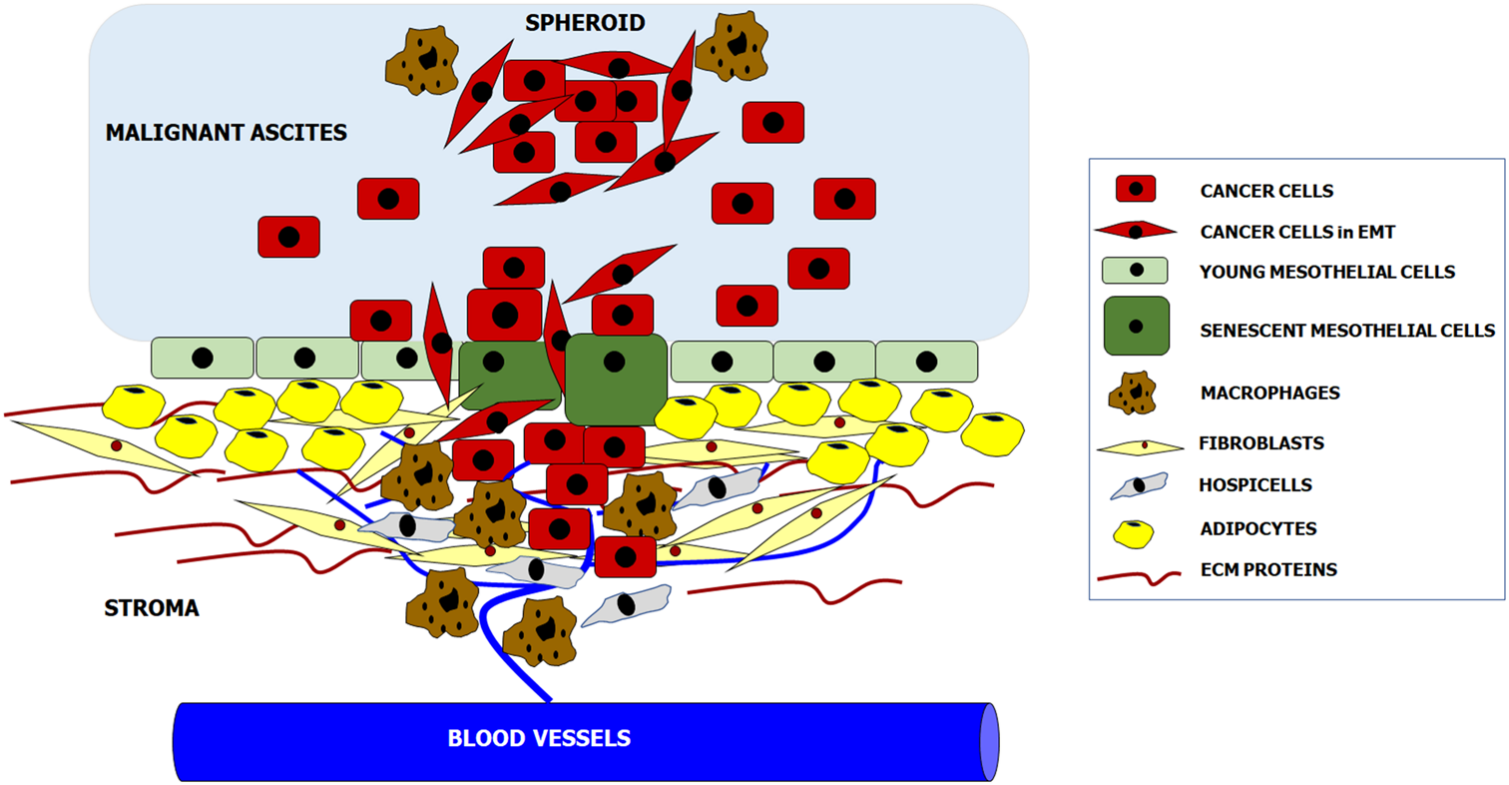
Cellular and acellular components creating metastatic niche within the peritoneal cavity. Complex molecular and biochemical background of these interactions is precisely delineated in the text

According to the newest knowledge, intraperitoneal formation of cancer metastases is orchestrated by reciprocal interactions between invading cancer cells and all populations of resident peritoneal cells. Some aspects of tumor progression, e.g., adhesion, are controlled primarily by specific cell types (mesothelial cells), whereas some other phenomena, e.g., proliferation and migration, are supported by almost all cell populations. In addition, normal cell-cancer cell interactions may proceed at four basic levels: upon their direct physical contact, through the paracrine activity of soluble factors released to the environment, and through reactions mediated by insoluble products of the cell secretome, e.g., ECM constituents. As was mentioned before, the function of both cancer cells and peritoneal cells may also be modulated by the presence and composition of malignant ascites (Fig. 1).

### Peritoneal mesothelial cells (PMCs)

The visceral and parietal surfaces of the peritoneal cavity are covered by a single layer of epithelial-like cells, i.e., mesothelial cells (PMCs). A unique feature of these cells is their dual, mesenchymal–epithelial characteristics. They originate as fibroblasts from the mesoderm, but their appearance and function resemble that of epithelial cells; hence, PMCs express intermediate filaments typical of both the mesoderm (vimentin) and epithelium (cytokeratins). Under certain stimuli, in particular TGF-β1, PMCs lose their cobblestone appearance and adopt a spindle-shaped morphology typical of cells undergoing the EMT [62].

Among all the fractions of cells forming the peritoneal cavity, PMCs are the largest, and thus, their role in the maintenance of intraperitoneal homeostasis is the most prominent [62]. Their involvement in cancer metastases was also studied most extensively among all types of normal peritoneal cells, which is probably due to the fact that they have direct interaction with inflowing cancer cells as the first. In this regard, however, there is still an ongoing debate as to the exact function of PMCs during the very first stages of intraperitoneal cancer progression.

According to a group of scientists, PMCs play a passive role as “the first line of defense”, whose disruption and concomitant penetration allows cancer cells to start interacting with the tissue stroma, in particular with the peritoneal fibroblasts and ECM constituents, and to freely disseminate [96, 97]. This assumption stems from the observation that biopsies of ovarian tumors that were present in the peritoneum did not contain mesothelial cells in close proximity to the proliferating cancer cells [98]. The authors of this statement explained the above by discussing the active behavior of cancer cells which generate myosin-related forces that push the mesothelial cells apart, which creates a mesothelium-free channel by which the malignant cells can reach the tissue stroma. Interestingly, in the image showing this situation in vivo, one can recognize cancer cells lying above the PMCs (not below—in the stroma), which indicates very initial stages of cancer progression, probably very close to their stable adhesion [98]. Nonetheless, the enthusiasts of the theory of the protective role of PMCs during intraperitoneal dissemination of ovarian cancer have provided more results confirming their reasoning, e.g., they showed that ovarian cancer cells attach more efficiently to the ECM than to PMCs [99]. Other authors observed, in turn, that PMCs inhibit ovarian cancer cell adhesion and invasion, while fibroblasts promote both phenomena [100]. In our opinion, it is worth noting, however, that the analysis of cancer cell adhesion to various cellular and acellular structures was based on quite a specific algorithm in which the efficiency of this process was estimated according to the mathematical difference between total adhesion of cancer cells to PMCs co-cultured with peritoneal fibroblasts and partial adhesion of these cells to PMCs alone.

On the other hand, there is a group of researchers, to which belongs also our team,favoring the scenario that PMCs do, indeed, support cancer cells in their attempts to colonize the peritoneal cavity. There is evidence that PMCs promote ovarian cancer cell adhesion via interactions between mesothelial cell surface fibronectin and cancer cell-derived α_5_β1 integrins [101] via the binding of mesothelial hyaluronic acid (HA) with its receptor, CD44, on the cancer cells [102], or via the activity of certain soluble agents released to the environment, e.g., lysophosphatidic acid (LPA) [103]. Moreover, several soluble factors of mesothelial origin have been found to stimulate other vital elements of ovarian cancer cell progression, including proliferation (CXCL8/IL-8, IL-6 [104]), migration (CXCL12/SDF-1 [105], HA [106]), and invasion (LPA [103]). Other PMC-derived agents are involved in remodeling of ECM (PAI-1 [107], u-PA [108]), angiogenesis (VEGF [86]), and EMT (TGF-β1 [109]).

Our own experiments designed to verify the role of PMCs in peritoneal ovarian cancer development have shown that the efficiency of ovarian cancer cell adhesion to the primary omental PMCs was considerably higher than to fibronectin and to fibroblasts. Moreover, ovarian cancer cells proliferated better in the presence of PMCs than in the presence of fibroblasts or fibronectin [110]. We also documented in experiments using immunocompromised mice that the rate at which ovarian tumors developed in the peritoneal cavity upon i.p. injection of mixtures of ovarian cancer cells together with PMCs was higher as compared with xenografts produced upon injection of cancer cells alone [110].

Two clashing ideas regarding the role of PMCs have resulted in a conceptual compromise that PMCs do indeed promote the early stages of ovarian cancer metastasis by TGF-β1/Smad-mediated up-regulation of fibronectin production. Blocking fibronectin production decreased the ability of ovarian cancer cells to adhere to PMCs and reduced their proliferation and invasion [111].

PMCs contribute to the progression of not only ovarian cancer cells. It has been evidenced that they also promote adhesion of colorectal and pancreatic cancer cells, albeit the molecular mechanisms underlying this interaction are different; namely, they involve the cooperation of cancer cell surface ligand CD43 and intercellular adhesion molecule-1 (ICAM-1) on the surface of the PMCs [112, 113]. The strength of cancer cell adherence has been recognized as being determined by local inflammation, in particular by the activity of IL-1β and TNFα [114, 115], and by oxidative stress [116, 117]. Unexpectedly, a very recent study showed in the case of colorectal and pancreatic cancer what has been challenged for ovarian cancer cells, i.e., protection of the peritoneal cavity by PMCs. It has been evidenced that colorectal (SW480) and pancreatic (PSN-1) cancer cells generated tumors in the mouse peritoneum cavity at higher dynamics when they were injected alone than in the presence of PMCs. Further in vitro studies showed that this effect could be associated with up-regulated secretion of soluble ICAM-1 (sICAM-1) by the PMCs which appeared to block the interaction of tumor-derived CD43 with its cell-bound counterpart in a competitive manner [118].

This last observation may suggest that the role of PMCs in peritoneal carcinomatosis may depend on the type of tumor cells. On the other hand, there is evidence that the contribution of PMCs may be determined by their replicative age. Interestingly, PMCs are the only type of cells originating from the peritoneum for whom both the triggers and the mechanisms of senescence as well as the resulting changes in gene expression and function have been well described. In brief, PMCs display poor proliferative capacity and fast entry into senescence, which closely resembles other kinds of epithelial cells. Senescence of PMCs proceeds in a telomere-independent fashion and is mediated by p16^INK4a^ [119]. What is of special importance for the potential clinical relevance of senescent PMCs is that their presence has been demonstrated in the omentum in vivo [120]. No less important is the observation that the senescence of PMCs is induced prematurely by malignant ascites-derived HGF and CXCL1/GRO-1 [121].

**Fig. 2:**
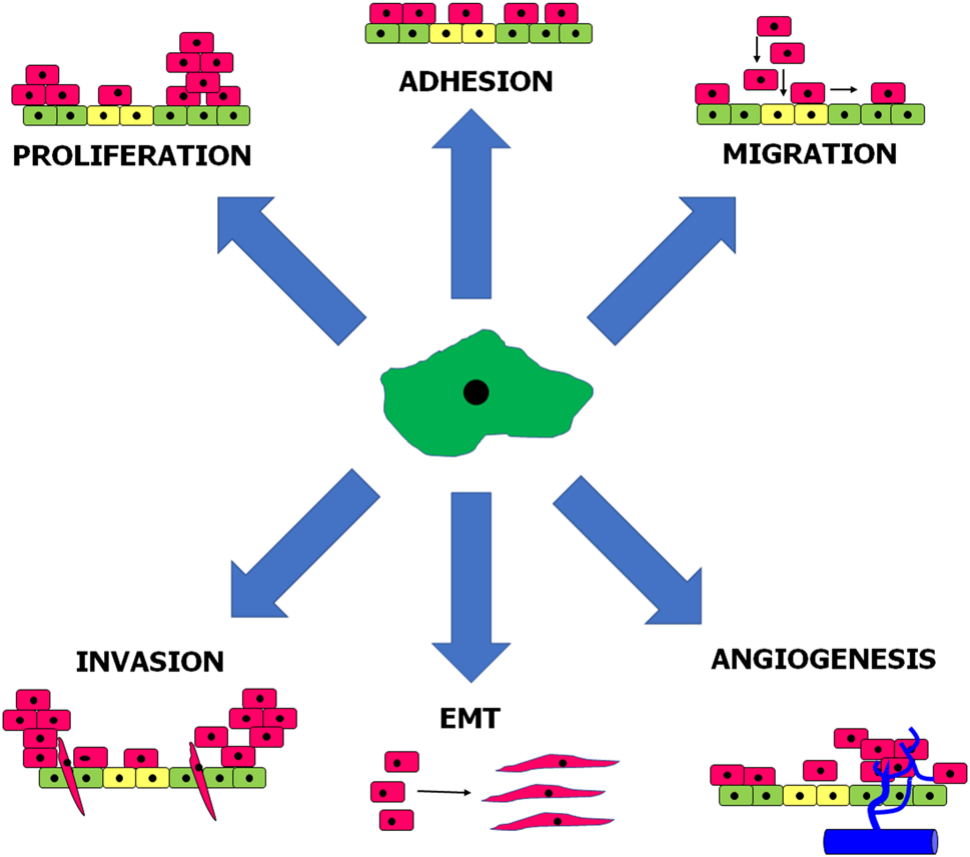
Elements of intraperitoneal cancer cell progression stimulated by senescent peritoneal mesothelial cells. Mediators and signaling pathways underlying these phenomena are discussed in the text

Experiments in vitro using primary, omental PMCs showed that senescent cells promote adhesion of ovarian [122], colorectal, and pancreatic cancer [123] cells much more effectively than young cells. As per ovarian cancer, the pro-adhesive capabilities of senescent PMCs have been linked with increased production of fibronectin by these cells and to concomitant augmented interactions between overexpressed fibronectin and α_5_β1 integrins on the surface of the cancer cells. Mechanistically, increased generation of fibronectin was related to an axis involving oxidative stress- and TGF-β1-dependent induction of p38 MAPK [122]. When it comes to cancers originating from the gastrointestinal tract, their improved adhesion to senescent PMCs resulted from p38 MAPK- and AP-1-dependent overproduction of surface ICAM-1 [123]. Furthermore, senescent PMCs appeared to stimulate proliferation, migration, and invasion of ovarian cancer cells in vitro, and this effect was related to both the activity of soluble agents released to the environment by these cells and to direct cell–cell contact (Fig. 2). In fact, senescent PMCs display well developed SASP, as they hypersecrete numerous proteins involved in cell replication, angiogenesis, inflammation, and ECM remodeling, and are known to promote certain elements of cancer cell progression. In the case of ovarian cancer cells, their motility was fueled by CXCL1/GRO-1, CXCL8/IL-8, IL-6, TGF-β1, and fibronectin [124]. Intervention studies allowed to discover that SASP present in senescent PMCs is elicited in a pathway engaging p38 MAPK and NF-κB [125]. Mice injected intraperitoneally with ovarian cancer cells combined with senescent PMCs formed tumors at higher dynamics as compared with those in which the tumors developed in the presence of young PMCs. Interestingly, when senescence and concomitant development of SASP were inhibited by neutralization of p38 MAPK, the rate at which the ovarian tumors progressed in vivo was significantly attenuated [124].

Apart from ovarian cancer, senescent PMCs also exert promoting activity towards colorectal tumors. Under in vitro conditions, they stimulated cancer cell proliferation (via IL-6), migration (via CXCL8/IL-8 and CCL2/MCP-1), and invasion (via IL-6, MMP-3 and uPA), and they triggered the EMT in a mechanism involving TGF-β1-dependent induction of Smad 2/3-Snail1 signaling. Experiments using a mouse xenograft model showed that they also stimulated the progression of intraperitoneal colorectal tumors, whose effect was partly associated with increased tumor neovascularization [125].

Cancer cell-type specificity of the pro-tumoral activity of senescent PMCs was confirmed in observations in which the PMCs were able to increase the adhesion [123] and migration of pancreatic cancer cells but simultaneously failed to stimulate their proliferation in vitro and tumor growth in vivo [125].

It is also worth noting that senescent PMCs may regulate the progression of ovarian cancer cells by reprogramming their secretory phenotype towards increased production of proangiogenic agents and the resulting stimulation of the angiogenic capabilities of the vascular endothelium. In this respect, an analysis of senescent PMCs’ secretome allowed to identify IL-6 and TGF-β1 as the mediators of their proangiogenic activity. At the transcriptional level, increased angiogenic behavior of endothelial cells subjected to cancer cells modified by senescent PMCs was regulated by HIF-1α, NF-κB/p50, and AP-1/c-Jun [87].

### Peritoneal fibroblasts (PFBs)

The submesothelial stroma of the peritoneal cavity is formed by PFBs and structural proteins secreted by these cells, including collagen, fibronectin, elastin, and vitronectin [126]. Once the cavity is colonized by a cancer, the PFBs start to act as CAFs supporting disease progression [127]. Simultaneously, it is not entirely clear what the exact origin of CAFs within the peritoneal tumors is; what is known for sure is that they do not derive from the cancer cells [128]. Classically, they were treated as resident cells that were activated by stimuli sent by the tumors [129]. A much newer theory states, however, that peritoneal CAFs may derive from PMCs in which cytoarchitectural changes, i.e., the development of a spindle-shaped appearance called the mesothelial–mesenchymal transition (MMT), are initiated in a reaction to the products of the cancer cell secretome [130]. A much earlier study proposed that myofibroblastic transdifferentiation of PMCs during peritoneal carcinomatosis may be elicited by TGF-β1 [131]. This scenario was confirmed in experiments on peritoneal gastric cancer metastases in which the expression of the fibroblast activation protein (FAP) was revealed in the mesothelial region of the majority of tumor specimens [132]. Activated PMCs displaying decreased expression of E-cadherin and increased expression of αSMA up-regulated the proliferation of gastric cancer cells either in a mechanism involving direct cell–cell contact or anchorage-independently [133]. Interestingly, the presence of PFBs with myofibroblastic characteristics seems to be a unique feature of malignant tumors, as these PFBs were not detected in the peritoneum of patients with benign ovarian lesions. In the case of cancer, their frequency expanded along with progression of the disease [134].

It is believed that malignant ascites play a potent role as the source of signals evoking the transdifferentiation of fibroblasts. The fluid contains high amounts of TGF-β1 and HGF, which are capable of promoting the MMT [80]. This concept was proved recently in a study in which malignant ascites-derived exosomes rich in TGF-β1 induced αSMA and FAP expression in PMCs and enhanced their motility [135].

Cancer-associated PFBs contribute to all processes underlying peritoneal carcinomatosis and there is no doubt, per analogy to PMCs, that their impact is clearly pro-tumoral. It has been found that they serve as an adhesive surface for cancer cell attachment in a mechanism involving β1-integrins [130].

PFBs become educated intraperitoneally to progress more efficiently in a paracrine manner by the cancer cells, in particular by TGF-β1. In normal cells, TGF-β1 activates one of its down-stream targets, Smad 2 [136], playing a significant role in the EMT/MMT phenomenon [137]. Experiments performed with a 3D culture model mimicking the omentum showed that activated PFBs supported both adhesion and invasion of the cancer cells in vitro, as well as tumor growth and metastasis in a mouse xenograft model. These activities were probably associated with overexpressed MMP-2 and HGF, as neutralization of these molecules markedly reduced tumor progression [136].

The activity of PFBs in the context of cancer progression is linked not only with TGF-β1 but also with TGF-α, whose expression is elevated in response to their co-culture with ovarian cancer cells. This effect is elicited by cancer cell-derived TNFα through the activation of NF-κB. TGF-α released by PFBs stimulates the development of peritoneal ovarian cancer metastasis in a mechanism engaging epidermal growth factor receptor (EGFR) signaling [138]. The activity of TNFα has also been linked with intraperitoneal spread of gastric cancer [139].

The universal pro-cancerous activity of PFBs was shown in studies conducted with pancreatic cancer cells whose migration and invasion were markedly increased in a co-culture system. The intraperitoneal spread of pancreatic cancer was also higher when the cancer cells were co-implanted into the mouse peritoneum together with PFBs [140]. Similar activity has been evidenced using mice xenografts generated by colorectal tumors [141].

The activity of PFBs also includes the modulation of intraperitoneal inflammatory responses, e.g., they are able to attract polymorphonuclear cells via products of their secretome, including CXCL1/GRO-1, CXCL8/IL-8, and G-CSF. PFBs’ ability to release those chemokines was regulated in a mechanism involving IL-1β [64]. Taking into account that IL-1β is constitutively produced by ovarian cancer cells [142], it is tempting to imagine that PFB-derived agents may contribute to mobilization and phenotypic alterations in the peritoneal macrophages infiltrating a tumor [143].

### Peritoneal adipocytes (PAs)

Recent years have provided a plethora of evidence that adipose tissue, in particular visceral obesity, significantly contributes to cancer development [144]. Accordingly, substantial progress has also been made in understanding the role of omental fat in intraperitoneal tumorigenesis. Studies using a two-dimensional co-culture system showed that omental adipocytes stimulate lipid (precisely: oleic acid) internalization by gastric cancer cells, the effect of which was followed by increased invasiveness of the latter. Intensified motility of the cancer cells was mediated by PI3K/Akt-related signaling and associated with the hyperactivity of MMP-2 [145]. Other research documented that ovarian cancer cells subjected to omental adipocytes display increased homing, migration, and invasion in mice, and that a potent role in this behavior was played by adipocyte-derived CXCL8/IL-8 [146].

Other evidence for adipocytes as energizers of cancerous tissue comes from experiments in which their co-culture with ovarian cancer cells resulted in increased lipolysis, whereas the cancer cells were characterized by increased β-oxidation. Moreover, omental metastases were characterized by higher expression of fatty acid-binding protein 4 (FABP4) than primary ovarian tumors [146]. Apart from the adipocytes, fueling peritoneal tumors in energy is also associated with the presence of omental adipose tissue-derived stem cells (ADSCs) which act in line with the “reverse Warburg effect” by providing lactose for the cancer cells and ATP generated in the glycolytic pathway [69].

The role of ADSCs in peritoneal carcinomatosis is, however, more complex, e.g., it has been reported that they are capable of promoting proliferation and invasion of pancreatic cancer cells. Mechanistically, this effect was associated with interactions between a pleiotropic chemokine, CXCL12/SDF1, released by the stem cells and its specific receptor, CXCR4, expressed on the surface of the cancer cells [147]. The pro-cancerous effect was also demonstrated utilizing ADSCs isolated from the omentum of patients with ovarian cancer which stimulated proliferation of the cancer cells in a co-culture system. Simultaneously, soluble agents released by the ADSCs to the conditioned medium supported the migration of cancer cells in vitro. A microarray evaluation revealed that the activity of ADSCs may be underlined by overexpressed genes coding for aggrecan, endocan, and matrilysin (MMP-7), all of which are involved in such aspects of cancer cell progression as adhesion, migration, angiogenesis, and ECM remodeling. Last but not least, ADSCs have been found to promote the resistance of cancer cells to chemo- (carboplatin and paclitaxel) and radiotherapy [69].

Interestingly, experiments using ADSCs isolated from mice showed that the tumorigenic activity of these cells is not a universal feature. Namely, the capacity to promote the development of intraperitoneal tumors was displayed by cells isolated from the visceral fat of obese animals, while cells obtained from lean subcutaneous adipose tissue lacked this activity. Another difference was the profile of pro-cancerous cytokines (e.g., IL-6 and CCL2/MCP-1) secreted by these two populations of cells [148].

### Peritoneal macrophages (PMs)

Although the general role of TAMs in tumorigenesis is well defined, the gene expression profiles of macrophages derived from various locations, e.g., the peritoneum, splenic red pulp, lung, or brain, revealed some diversity, thus implying that these cells’ anatomical localization may determine their functional phenotype [149]. When it comes to the peritoneal cavity, milky spots are an important reservoir of PMs [150], whose primary role within this structure is associated with the absorption and elimination of bacteria and debris from the peritoneum [151]. As per peritoneal carcinomatosis, the significance of PMs is wide [152]. Primarily, they contribute to the homing of cancer cells and fulfill this role upon their mobilization from the blood by tumor-derived chemoattractants (e.g., CCL2/MCP-1, IL-6, MIF, and CSF-1) and differentiation into TAMs [153]. Moreover, PMs play a role in the formation of spheroids during the early transcoelomic metastasis of ovarian cancer [154].

As the other types of TAMs, e.g., those accompanying breast tumors, the cells infiltrating ovarian cancer transform into the pro-cancerous M2 phenotype which is driven by factors present in malignant ascites. Such ascites-related activity may be mediated by IL-6 and IL-10, whose level positively correlated with the expression of the surface marker of M2 cells, i.e., CD163 [155]. These observations clearly pointed to the presence of mixed (M1/M2) populations of PMs in the malignant ascites. Similar conclusions were provided by other authors, who additionally revealed that the survival of patients with ovarian cancer depends on the ratio between anti-tumoral M1 and pro-tumoral M2 cells [156].

M2 polarization of macrophages may also occur independently in the presence of ascitic fluid. Research on PMs co-cultured with gastric cancer showed that they adopted the M2 phenotype in response to soluble agents released by the latter [157]. This effect coincided with the phosphorylation of STAT3, which is currently considered as one of the key molecules responsible for the development of the macrophage M2 phenotype [158]. The functional polarization of PMs influences invasive gastric cancer cell behavior, as they support by the M2 macrophages resulted in improved proliferation and accelerated tumor growth in the xenograft model [157]. Other signaling pathways activated in the cancer cells (here ovarian cancer) by PMs include JNK and NF-κB pathways. Their activation coincided with up-regulated expression of genes coding for the extracellular matrix metalloproteinase inducer (EMMPRIN) and increased invasiveness [159]. In addition, experiments on mice showed that either ascite formation or peritoneal metastasis could be prevented by depletion of neutrophils or NK cells but not PMs, which may indicate that the presence of the activity of those cells may, to some extent, be a limiting factor for effective peritoneal carcinomatosis [160].

An important role of PMs concerns the modulation of immune reactions within the peritoneal cavity, e.g., they are the primary source of CCL22, which is highly involved in the recruitment of immunosuppressive Treg cells into tumors [161]. It has been found that this chemokine’s level in malignant ascites from patients with ovarian cancer was significantly higher than in patients with benign tumors-serous cystadenoma. Moreover, patients with advanced stages of the disease, which is usually associated with the peritoneal burden, also had a markedly elevated plasma level of CCL22 as compared with patients in early stages [162]. Significantly, the vicious circle closes when Treg cells attracted to the tumor activate a retrograde response in which they stimulate the PMs to M2 polarization through their own IL-4, IL-10, and IL-13 [163].

Another role of PMs is their contribution to intraperitoneal angiogenesis, as they produce various proangiogenic stimuli, including VEGF, MMP-1, and amphiregulin [157]. Experiments using mice peritoneal macrophages revealed that their proangiogenic potential is elicited particularly in hypoxic conditions. When conditioned media harvested from PMs were mixed with Matrigel and injected into mice, they yielded significantly greater expansion of microvessels as compared with Matrigel plugs containing supernatants from macrophages maintained in normoxic conditions. Mechanistically, this effect was mediated by HIF-1α, whose nuclear translocation was responsible for the increased concentration of numerous proangiogenic stimuli (IL-6, IL-12, CCL2/MCP-1, CCL5, CXCL8/IL-8, and VEGF) in conditioned media from PMs kept under hypoxia [164].

### Peritoneal endothelial cells (PECs)

Endothelial cells infiltrate the peritoneum along with macrophages in the vicinity of tumor implants. There is evidence that the mobilization of endothelial cells towards their angiogenic reactions (proliferation, migration, and tube formation) results from cooperative signals sent by cancer cells and PMs. The co-culture of PMs with ovarian cancer cells up-regulated the production of CXCL8/IL-8 by the latter, which was responsible for increased migration of endothelial cells and the formation of tubular structures in response to conditioned media from these co-cultures (as compared with media harvested from separate cultures of cancer cells or PMs). Mechanistically, this effect was linked with the activity of NF-κB [165].

Mobilization followed by increased mobility of endothelial cells is also orchestrated by the products of the secretome of cancer cells, PMCs, and PFBs, e.g., ovarian cancer cells secrete high amounts of CXCL1/GRO-1, CXCL8/IL-8, IL-6, HGF, and VEGF [87], whereas PMCs generate constitutively CXCL1/GRO-1, CXCL12/SDF1, bFGF, MMP-2, MMP-9, and VEGF [124, 125]. Proof for the angiogenic potential of agents produced by ovarian cancer cells derives from experiments in which both the proliferation and migration of endothelial cells was stimulated by a conditioned medium of cancerous origin [87]. As per individual proteins, the proliferation, migration, and tube formation of endothelial cells bearing CXCR1/2 chemokine receptors were increased in response to CXCL8/IL-8 and CXCL1/GRO-1 produced by ovarian cancer cells in a mechanism involving MMP-1-protease-activated receptor-1 (PAR1) activation. When cell-penetrating pepducin, X1/2pal-i3, targeting the third intracellular loop of CXCR1 and CXCR2 was introduced angiogenic endothelial cell behavior in mice xenografts significantly declined [166]. The formation of tubular structures by endothelial cells in vitro was also effectively prevented when the conditioned medium generated by PMCs was pre-incubated with a VEGF neutralizing antibody [167].

Cancer cells and endothelial cells may also interact under certain circumstances in such a way that progression of the disease becomes limited. This conclusion stems from research on ovarian cancer cells engineered to express a gene for vasohibin-1 (VASH1) that is normally expressed by endothelial cells in response to angiogenic stimuli and inhibits these cells’ motility autocrinally in a negative feedback mechanism [168]. The release of VASH1 by cancer cells inhibited the growth of endothelial cells in vitro, and tumor neovascularization and expansion in mice in vivo [169].

### Peritoneal hospicells

Bone-marrow mesenchymal stem cells (BM-MSC) are attracted to various anatomical locations where they actively contribute to cancer development. These original mesenchymal stem cells (CD9, CD10, CD29, CD146, CD166, and HLA-1) were first described by Rafii and colleagues in malignant ascites from patients with ovarian cancer and were called “hospicells”. Their presence was initially linked with the chemoresistance of ovarian tumors to platin and taxans [170]. This effect is probably associated with hospicells’ ability to produce insulin-like growth factor 1 (IGF-1) which controls the expression of various ATP-binding cassette (ABC) genes (MDR1, MRP1, MRP2, MRP3, MRP5, and BCRP) utilizing PI3-kinase, MEK, and JAK2/STAT3 signaling routes [171].

Further experiments revealed that the significance of hospicells is much broader. It has been demonstrated that their co-injection with ovarian cancer cells into the mouse peritoneal cavity enhanced tumor growth and accumulation of ascites. Lesions that developed by the co-injection of hospicells and ovarian cancer cells displayed improved vascularization, which suggested the proangiogenic capabilities of these cells [172]. This assumption was confirmed by further experiments in which the bidirectional migration of hospicells towards endothelial cells and vice versa was demonstrated. In addition, hospicells synergized with ovarian cancer cells to secrete increased amounts of proangiogenic VEGF, IL-6, and CXCL8/IL-8 [173].

Another activity of hospicells is immunosuppression, as they were found to inhibit the proliferation of CD4(+) and CD8(+) T-cells as well as to restrict the secretion of cytokines by these cells [174]. They are also capable of attracting PMs and of converting them into the M2 phenotype [173].

## Conclusions and perspectives

Taken together, the knowledge about cellular and molecular mechanisms underlying the intraperitoneal development of cancer metastases is very well established. There are, however, some issues that need further investigations. The most important is, in our opinion, the role of normal peritoneal cells, in particular stromal cells, in cancer recurrence. Further examinations are also necessary to verify to what extent certain manipulations within the phenotypic features of peritoneal cells, e.g., those resulting from targeting some signaling pathways associated with senescence of PMCs may effectively inhibit or postpone the development of various pro-tumoral features of the peritoneum. Last but not least, it also needs to be explained to what extent normal peritoneal cells are genetically and functionally changed in response to systemic and intraperitoneal chemotherapy, and how these drug-modified cells behave in relation to residual or recurrent disease.
